# Blocking mineralocorticoid receptor with spironolactone may have a wide range of therapeutic actions in severe COVID-19 disease

**DOI:** 10.1186/s13054-020-03055-6

**Published:** 2020-06-08

**Authors:** Lucas Liaudet, Csaba Szabo

**Affiliations:** 1 The Service of Adult Intensive Care Medicine, University Hospital Medical Center, Rue du Bugnon 46, 1011 Lausanne, Switzerland; 2 The Department of Pharmacology, Faculty of Science and Medicine, University of Fribourg, 1700 Fribourg, Switzerland

The pulmonary renin angiotensin (Ang) system (RAS) comprises two pathways whose balance is important for lung homeostasis. Endothelial ACE generates Ang II, acting on AT1 receptors to promote vasoconstriction and pro-inflammatory effects, whereas epithelial ACE2 cleaves Ang II into Ang1–7, acting on the Mas receptor to exert vasodilatory and anti-inflammatory effects. A shift towards predominant ACE-dependent Ang II formation has been postulated as an important pathophysiological mechanism in various forms of ARDS [1].

SARS-CoV-2 uses lung ACE2 as its cellular receptor, resulting in ACE2 degradation and ACE/ACE2 imbalance, which could drive Ang II-mediated vascular inflammation and lung injury in severe COVID-19 disease [1]. Furthermore, Ang II induces the release of aldosterone, which can promote further vascular damage via mineralocorticoid receptor (MR) activation [2]. Aldosterone also exerts multiple actions on immune cells, which express the MR [3]. MR activation polarizes macrophages towards the M1 pro-inflammatory phenotype. In lymphocytes, MR activation promotes the differentiation of pro-inflammatory Th17 CD4^+^ cells and of cytotoxic IFNγ^+^-CD8^+^ T cells (Fig. 1), indicating that MR activation in immune cells promotes a hyperinflammatory profile [3]. It is particularly noticeable that Th17 T cells increase and high CD8^+^ cells cytotoxicity have been proposed to be involved in the hyperinflammatory state characterizing COVID-19 ARDS [4].

**Fig. 1:**
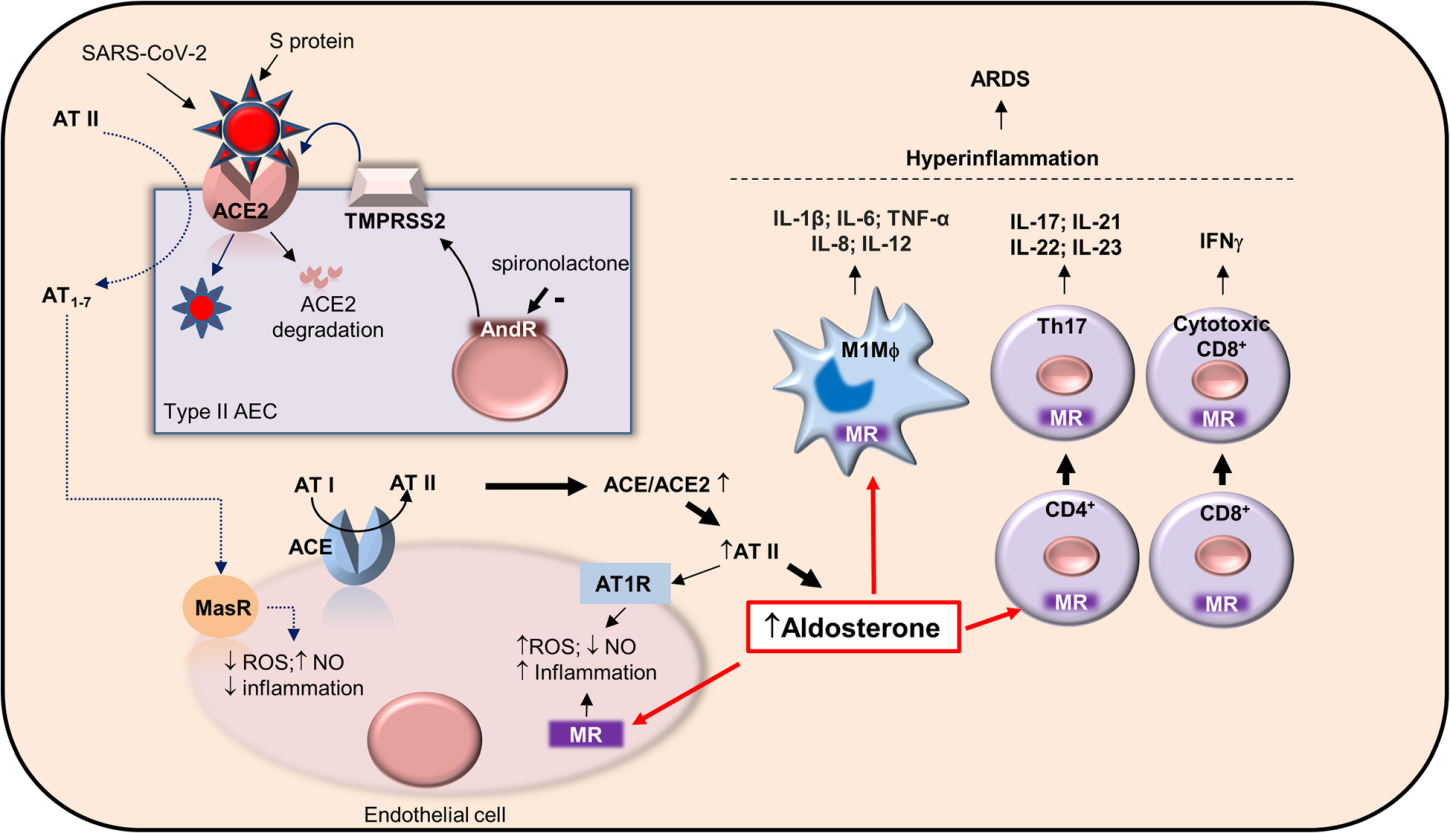
Sites of potential pharmacological actions of spironolactone in COVID-19 ARDS. SARS-CoV-2 infects type II alveolar epithelial cells (type II AEC) via interaction between its spike S protein and ACE2 receptor, promoting internalization and degradation of ACE2 and pulmonary ACE/ACE2 imbalance. In turn, the degradation of angiotensin II (AT II) into angiotensin 1–7 (AT_1–7_) is prevented (dotted lines), reducing anti-inflammatory signaling through the Mas receptor (MasR), and promoting pro-inflammatory AT II signaling through the angiotensin receptor type I (AT1R) in vascular endothelial cells. Increased AT II results in aldosterone formation, which signals through the mineralocorticoid receptor (MR), leading to vascular inflammation and immune cells activation. MR activation polarizes macrophages towards the M1 pro-inflammatory phenotype (M1Mϕ), favors CD4^+^ lymphocytes differentiation towards pro-inflammatory Th17 cells, and induces cytotoxic IFNγ^+^-CD8^+^ lymphocytes. The development of a hyper-inflammatory state may trigger ARDS. MR inhibition with spironolactone may interrupt the deleterious actions of aldosterone. Furthermore, via its anti-androgenic effects, spironolactone may decrease the expression of TMPRSS2, a serine protease priming the S protein for its interaction with ACE2. Abbreviations: ACE, angiotensin-converting enzyme; AndR, androgen receptor; AT I, angiotensin I; AT II, angiotensin II; AT_1–7_, angiotensin 1–7; AT1R, angiotensin receptor type I; MR, mineralocorticoid receptor; MasR, Mas receptor; NO, nitric oxide; ROS, reactive oxygen species; TMPRSS2, transmembrane serine protease 2

Dysregulated RAS signaling with enhanced aldosterone-mediated MR activation could represent an important link between SARS-CoV-2/ACE2 interaction and inflammatory lung injury, suggesting an interesting therapeutic potential of RAS inhibitors [1] and in particular MR antagonists. However, it has been claimed that RAS inhibitors could enhance ACE2 expression, which might represent a possible drawback of this therapeutic strategy, as this might influence SARS-CoV-2 infectivity [1]. Importantly, in contrast to other RAS inhibitors, the MR antagonist spironolactone also possesses significant anti-androgenic actions [5]. Such effects may be particularly useful in the context of SARS-CoV-2 infection, by inhibiting the androgen-dependent expression of TMPRSS2, a transmembrane protease crucial for viral entry through its priming effect on the viral S protein [5]. Therefore, by its dual actions as an MR antagonist and an androgenic inhibitor, spironolactone might provide significant benefits in COVID-19 ARDS. Naturally, the primary action of spironolactone (reduction of pulmonary edema) would also be beneficial in COVID-19 ARDS. Thus, we hypothesize that through its combined pharmacological actions, spironolactone may provide therapeutic benefit, when applied in the later stage of COVID-19 ARDS. Clinical trials may be warranted to evaluate its therapeutic potential.

## Data Availability

NA
